# Surgical thrombectomy for iliofemoral deep vein thrombosis: Patient outcomes at 8.5 years

**DOI:** 10.1371/journal.pone.0235003

**Published:** 2020-06-18

**Authors:** Dominic Mühlberger, Martin Wenkel, Georg Papapostolou, Achim Mumme, Markus Stücker, Stefanie Reich-Schupke, Thomas Hummel

**Affiliations:** 1 Department of Vascular Surgery, St. Josef Hospital Bochum, Katholisches Klinikum Bochum, Ruhr University Bochum, Bochum, Germany; 2 Vein Center of the Departments of Dermatology and Vascular Surgery, Katholisches Klinikum Bochum, Bochum, Germany; 3 Department of Dermatology, St. Josef Hospital Bochum, Katholisches Klinikum Bochum, Ruhr University Bochum, Bochum, Germany; University Magna Graecia of Catanzaro, ITALY

## Abstract

**Introduction:**

Deep vein thrombosis (DVT) is a frequent burden and a post-thrombotic syndrome (PTS) can be a serious long-term consequence. Iliofemoral DVT should be associated with severe forms of PTS. Therefore an early thrombus removal has been recommended in specific conditions. The aim of this study was to find out both, the long-term results after surgical thrombectomy of iliofemoral DVT in respect of the development of PTS as well as the venous hemodynamics after surgery concerning venous reflux and venous obstruction.

**Methods:**

Sixty-seven patients who underwent surgical thrombectomy between the years 2000 and 2014 were included in this study; iliofemoral DVT was present in 52 of these patients. 35 patients could be reinvestigated after a mean follow-up of 8.5 years. CEAP (Clinical-Etiological-Anatomical-Pathophysiological) and Villalta scores were recorded in order to describe and assess PTS. Follow-up examinations included a detailed duplex mapping. Venous hemodynamics were measured by digital photoplethysmography and venous occlusion plethysmography.

**Results:**

The primary patency rate of the iliofemoral segment was 88% after 8.5 years. 48% of all patients showed reflux in deep vein segments. Mild or moderate PTS occurred in 57% of all patients. Notably, there was no patient with an active ulcer or severe PTS. The mean venous outflow volume of all patients in the treated legs was 66.1 ml/100ml/min and significantly less than in the controlled contralateral non-treated legs (p<0.05). The mean venous refilling time was 16.3 seconds, while the mean value of the non-treated contralateral legs was 25.6 seconds and therefore significantly higher (p<0.05).

**Conclusion:**

Even though venous hemodynamics are significantly inferior in the treated legs, this study demonstrates excellent patency rates and good clinical outcome after surgical thrombectomy of iliofemoral veins.

## Introduction

Deep vein thrombosis (DVT) is a frequent burden, as the incidence is about 300/100000 patients per year [1]. Whereas a pulmonary embolism is the most critical early complication, the development of a chronic thrombo-embolic pulmonary hypertension (CTEPH) or a post-thrombotic syndrome (PTS) can be serious long term consequences as well [2]. Venous ulcers and a “so-called” venous claudication are the most notable symptoms of severe PTS and can significantly reduce quality of life [3]. In particular, iliofemoral DVT is associated with severe forms of PTS [4–7]. Therefore, an early thrombus removal has been recommended in specific conditions. Currently, endovenous methods and surgical thrombectomy are available treatment options [8]. Especially, young patients with an acceptable life expectancy with an acute, symptomatic iliofemoral DVT and a thrombus age less than 14 days are supposed to benefit from early thrombus removal strategies [6]. Endovascular treatment with pharmacological or pharmacomechanical devices has been recommended as first-line therapy [6]. Nevertheless, there was no advantage for the additional use of pharmacomechanical catheter-directed thrombolysis compared with anticoagulation alone with respect to the prevention of PTS in a recently published study [9]. Restrictively, this could be due to the fact that patients with iliofemoral as well as femoropopliteal DVT have been included in this trial [9–11]. Thus, a subgroup analysis of this study shows a benefit in the reduction of severe PTS for the use of pharmacomechanical catheter-directed thrombolysis, limited to patients with an iliofemoral DVT [11]. However, there have been demonstrated good results for surgical thrombectomy of iliofemoral DVT in terms of prevention of PTS as well [1, 8, 12–17]. Even though duplex ultrasound mapping is the gold standard in the diagnosis of venous diseases including PTS [18], other non-invasive diagnostic methods e.g. photoplethysmography, venous occlusion plethysmography or air plethysmography are available [19]. Especially after surgical thrombectomy, there exist only few data concerning the hemodynamic results diagnosed by photoplethysmography and venous occlusion air plethysmography, even though their diagnostic outcome may predict ulcer healing and recurrence [12, 20, 21]. Hence, the aim of this study was to elucidate both the long-term results after surgical thrombectomy of iliofemoral DVT with respect to the development of PTS and the venous hemodynamics concerning venous reflux and venous obstruction.

## Material and methods

The study was approved by the Ethics Committee (Registry Number 16–5819 from 30.08.2016) of the medical faculty of the Ruhr University Bochum. Inclusion criteria were previous iliofemoral DVT treated by venous thrombectomy and age over 18 years. We defined the following exclusion criteria: septic thrombosis, cancer, pregnancy-associated DVT, atresia of deep veins, phlegmasia cerulea dolens or iliofemoral DVT due to mechanical reasons, e.g. catheter-associated DVT related to a cardiac examination or venous catheters. These exclusion criteria were defined in order to get a homogenous study population, who was surgically treated due to an “unprovoked” iliofemoral DVT.

We treated 108 patients by venous thrombectomy between the years 2000 and 2014 in our department. Due to incomplete medical data 19 patients could not be included in the study and 22 patients did not fulfill the inclusion and/or exclusion criteria. Therefore, 67 patients could be included into the study. These 67 patients presented with an iliofemoral DVT, which was diagnosed by duplex ultrasound. There were 52 patients with an extended ascending DVT from the lower leg to iliofemoral vein segments. The inferior caval vein was involved in nine of these patients. Furthermore, there were 15 patients with an isolated descending DVT of the iliofemoral vein segments. In these cases the femoral vein and downstream veins were free of thrombus. One of these patients had an involvement of the inferior caval vein (Table 1).

**Table 1:** Localization and prolongation of DVT.

Thrombus localization	Number of patients
DVT from the leg to iliofemoral vein segments	43
DVT from the leg to inferior caval vein	9
Isolated DVT of iliofemoral veins (without DVT of the femoral vein)	14
Isolated DVT of iliofemoral veins and inferior caval vein (without DVT of the femoral vein)	1

DVT (deep vein thrombosis)

All patients gave informed written consent prior to surgery. Venous thrombectomy was performed by a standardized operating procedure. The common femoral vein was prepared by a longitudinal skin incision at the groin. After clamping the vein, it was opened longitudinally. Afterwards, the venous thrombectomy of the iliac veins or the inferior caval vein was performed by multiple Fogarty maneuvers under fluoroscopy. If the inferior caval vein was involved, a “blocking” Fogarty catheter was placed into the inferior caval vein proximal to the occlusion, by an additional venotomy of the contralateral common femoral vein, in order to prevent pulmonary embolism events during the venous thrombectomy maneuvers. Surgery was performed under anticoagulation with 5000 IU of heparin and a positive end expiratory pressure of 20 mmHg. Furthermore, patients were placed in reverse Trendelenburg position. Bimanual compression of the leg was used to remove thrombus clots of the leg. Subsequently, an ascending phlebography was routinely performed and, if necessary, a venous stent was placed in the iliac veins. In order to prevent a recurrent thrombosis, an arteriovenous fistula was constructed. Postoperative controls were made by duplex sonography. Compression therapy with a pressure between 23–32 mmHg and anticoagulation were recommended for at least six months. The arteriovenous fistula was closed three months after the procedure.

**Fig 1:**
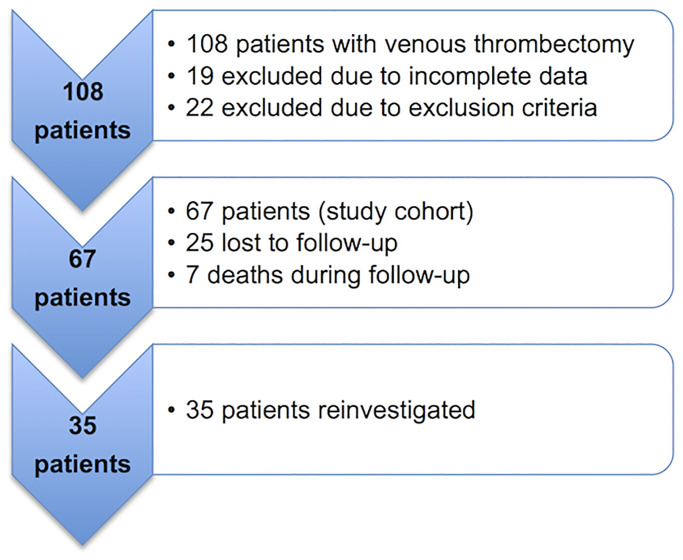
Patient recruitment. A total of 67 patients were included. 35 patients could be reinvestigated after a mean follow-up of 102 months (minimum 26,6 maximum 197,6).

We investigated 35 patients, as 25 patients were lost to follow up and 7 patients died during the follow up period. All follow-up patients were included with written informed consent for reinvestigation. They were invited for an additional clinical and physical examination (Fig 1).

The postoperative investigations were performed between Jan 1, 2016 and Dec 31, 2017. The mean follow-up time was 102 months (minimum 26.6 months, maximum 197.6 months, standard deviation 57.7 median 83.5). PTS was described and assessed by the Villalta score and the CEAP (Clinical-Etiological-Anatomical-Pathophysiological) score. Furthermore, a complete duplex sonography of the inferior caval vein, external iliac vein, common femoral vein, deep femoral vein, femoral vein, popliteal vein, anterior and posterior tibial veins and the fibular veins was part of the reinvestigation. Additionally, the great and small saphenous veins were analyzed. An independent investigator, who was not involved in the prior surgical procedure, performed the duplex scan. Duplex scans of the inferior caval vein, external iliac and common femoral vein were performed in a supine position. All other duplex scans were done in a standing position. Additionally, a photoplethysmography and a venous occlusion air plethysmography were performed, as previously described [5, 19]. The most important parameter of the photoplethysmography is the “venous refilling time” measured in seconds, which is a marker of venous reflux. A value <25 s is classified as pathological. The most important value of the venous occlusion plethysmography is the venous outflow volume in ml/100ml/min, as an indirect marker of iliofemoral obstruction [5]. In case of an outflow obstruction in the iliac veins, the target outflow volume is < 40–80 ml/min. Statistic analysis was performed by Microsoft Excel 2010 (descriptive statistics and students t test; data are presented as frequency distributions and percentages. Kaplan Maier calculation was used for patency). P< 0.05 was regarded significant.

## Results

Nearly half of the study population was male, the other half female. The mean age at the time of surgical treatment was 51 years. There were treated 19 right and 48 left legs. The mean thrombus age at the time of surgery was 3.7 days. Only 12 of 67 patients did not receive an arteriovenous fistula. Primary success rate was 93% with open iliofemoral vessels. Due to the fact that 25 patients were lost to follow-up and seven patients died, we had a follow-up rate of 52%.

The mean age of these 35 patients at the time of surgery was 48 years. There were 18 female and 17 male patients with 11 right and 24 left treated legs. The mean thrombus age at the time of surgery was 3.5 days. An arteriovenous fistula was prepared in 29 patients and venous stents were implanted in ten patients, whereas in only one case a transluminal angioplasty was performed. The mean follow-up time was 102 months (minimum 26.6 maximum 197.6). There was no perioperative mortality. The mean length of stay was 8 days. The mean operating time was 113 minutes. We had no postoperative bleeding or perioperative death. There were four (6%) patients with a postoperative wound infection and two (3%) patients with a postoperative seroma.

### Results after a mean follow-up of 102 month

#### Patency of iliofemoral vein segments and duplex ultrasound examination

**Fig 2:**
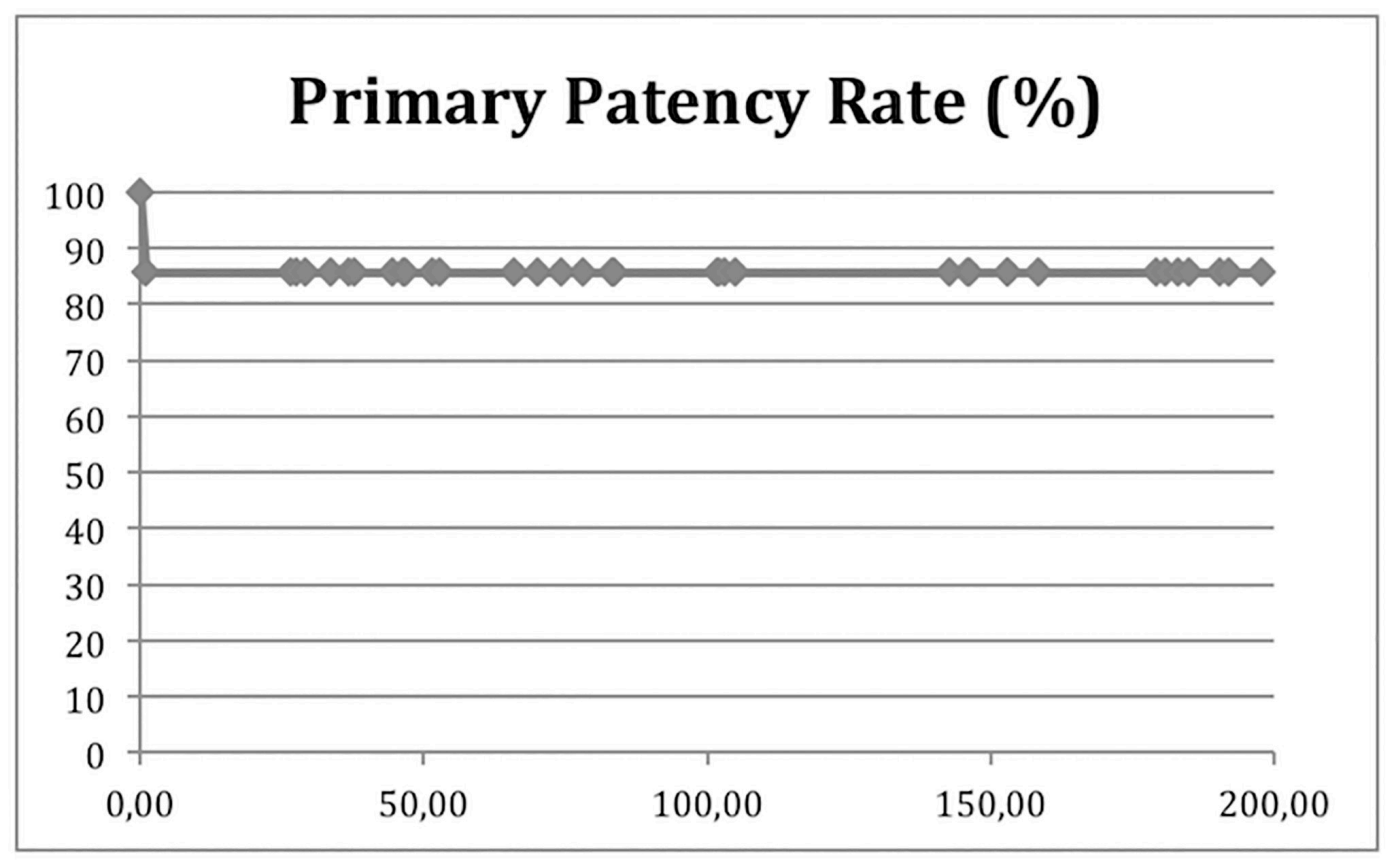
Patency rates. Kaplan-Meier estimation for primary patency rate in patients with iliofemoral DVT after surgical venous thrombectomy (horizontal axis: month after surgery, vertical axis percentage of open iliofemoral segments).

An “incomplete” venous recanalization, with a residual thrombus, was seen in five patients in the postsurgical examination. There were four patients with an occlusion of the iliac or common femoral vein. One patient had an occlusion of the femoral vein with an open deep femoral and common femoral vein. Nevertheless, these results show patency rates of 88% (31/35) after 8,5 years (Fig 2).

Postthrombotic alterations, e.g. septum or structural vein wall irregularities have been found in eight patients. Regarding reflux, there were three patients with reflux in the iliac veins and 14 patients with reflux in the deep veins distally to the common femoral vein. Most of these patients demonstrated reflux in the femoral or popliteal vein. Concerning reflux of the superficial veins, there were four patients with an insufficiency of the terminal valve of the great saphenous vein, one patient with reflux in the great saphenous vein above the knee and two patients with an incomplete, segmental insufficiency of the great saphenous vein. Only one patient demonstrated reflux at the small saphenous vein to the middle of the calf on the previously operated leg.

#### CEAP and villalta score

There was no patient with an active ulcer. Only one patient had a healed ulcer (C5) on the “formerly treated” leg and another one had a healed ulcer on the contralateral leg. Regarding the CEAP classification there were twelve patients with C0, ten patients with C1 and only two patients with visible varicose veins (C2). There were four patients with a pretibial edema (C3) and six patients had skin changes (C4) (Table 2).

**Table 2:** CEAP classification.

C0	C1	C2	C3	C4	C5	C6
12 (34%)	10 (29%)	2 (6%)	4 (11%)	6 (17%)	1 (3%)	0 (0%)

Number of patients according to the CEAP (Clinical-Etiological-Anatomical-Pathophysiological) classification after a mean follow up of 8.5 years (percentage in brackets)

Regarding the Villalta score there was no patient with a severe post thrombotic syndrome (Score > 15) [2]. There were four patients with moderate PTS (score between 10–14) and 16 patients with mild PTS (score between 5–9). Interestingly, 15 patients had a Villalta score below 5 (Table 3).

**Table 3:** Villalta score.

Villalta < 5 (no PTS)	Villalta 5–9 (mild PTS)	Villalta 10–14 (moderate PTS)	Villalta > 15 or ulcer (severe PTS)
15 (43%)	16 (46%)	4 (11%)	0 (0%)

Number of patients according to Villalta Score after a mean follow-up of 8.5 years (percentage in brackets)

#### Venous occlusion- and photoplethysmography

Both examinations were performed on 32 patients. Routinely, these examinations were performed on both legs. Regarding the venous occlusion plethysmography, the mean venous outflow volume of all patients in the treated legs was 66.1 ml/100ml/min with a standard deviation of 29.7. Six patients had a pathological value <40 ml/100ml/min. Compared to duplex ultrasound examinations, two of these patients had an incomplete recanalization of the iliofemoral vein and one patient demonstrated a complete obstruction of the common femoral vein. Furthermore, another patient had an obstruction and one demonstrated severe irregularities of the femoral vein at the thigh. The mean outflow volume in the not-treated “healthy” legs was 81.0 ml/100ml/min with a standard deviation of 32.6 and was significantly higher (p<0.05).

Regarding the photoplethysmography, the mean venous refilling time of the treated legs was 16.3 ± 12.8 s. There were 24 patients with a pathological venous filling time less than 25 seconds. Furthermore 14 patients had a highly reduced venous refilling time less than 10 seconds, which may indicate severe reflux. Concerning the duplex ultrasound examinations, ten of these 14 patients had reflux in the deep venous system too. Another two patients had severe post thrombotic changes and only two did not show any structural changes. The mean venous refilling time of the non-treated legs was 25.6 seconds with a standard deviation of 12.5, which is significantly higher compared with the treated legs (Table 4).

**Table 4:** Hemodynamic outcomes.

	treated legs	non-treated legs	p
Mean venous outflow (ml/100ml/min)	66.1	81.0	< 0.05
Mean venous refilling time (seconds)	16.3	25.6	< 0.05

Mean venous outflow detected by occlusion plethysmography and mean venous refilling time diagnosed by photoplethysmography after a mean follow-up of 8.5 years (S1 Table)

## Discussion

The primary patency rate of 88% in this study is comparable to former studies. To the best of our knowledge, the mean follow-up of 102 month represents one of the longest follow-up periods in the literature so far. Only Plate et al reported 10 year results prospectively and a patency rate of 83% after surgical thrombectomy [12]. These data have been confirmed by others and by our previously published results [1, 13, 16]. For example, Hölper et al reported primary and secondary patency rates after a mean follow-up of 64 month of 74% and 84% respectively [1] and Wagenhäuser et al described primary patency rates of 89% after a mean follow-up of 63 month [13]. Despite these excellent patency rates, we identified four patients with an occlusion of the iliac- or common femoral vein and one patient demonstrated an occlusion of the femoral vein with a patent iliofemoral segment. This corresponds to an occlusion rate of 11%. Retrospectively, all five patients had an early re-occlusion within the first postoperative years. This may indicate, that once a patient is treated successfully, the veins will remain patent. Other authors observed lower occlusion rates of 8.3% [13]. However, the initially enrolled 67 patients had a 48% loss of follow-up, which is another limitation of this study. Eminently, it cannot be excluded that the patients lost to follow-up affected the findings of our study. However, this study is nearly descriptive. Thus, there is no statistical comparison of two alternative therapeutic approaches. Therefore we are unable to make use of a statistical compensation for the lost to follow-up patients. Nevertheless, comparable studies in the last years had a smaller number of patients [1, 13, 15, 17].

Nearly the half of our treated patients had reflux in the deep venous system. Plate et al reported reflux rates of 33% in the femoral vein and 78% in the popliteal level after surgical thrombectomy [12]. Severe reflux, diagnosed by photoplethysmography, occurred in 40% of all patients and was mostly confirmed by duplex sonography. However, two patients had a normal duplex ultrasound mapping even though they demonstrate a venous refilling time <10 s. In general, the venous refilling time, as a marker of reflux, was significantly reduced in the formerly treated legs, when compared to the contralateral non-treated legs. Nevertheless, surgical thrombectomy should significantly reduce venous reflux, when compared to a conservative treatment with anticoagulation alone [8]. Unfortunately, due to the retrospective design of this study, we had no comparative “conservative or endovenous” group, which is a serious limitation of this study. According to the venous occlusion plethysmography all patients with a pathological measurement had a re-occlusion of the iliofemoral segment or the femoral vein respectively. As expected, the venous outflow, as a marker of obstruction, was higher in healthy legs, when compared to treated legs. Nevertheless, the mean venous outflow in the treated legs of our patients was 66 ml/min/100ml, which represents a physiological value. Plate et al reported similar results [12] after surgical thrombectomy. Due to the retrospective character of this study, photoplethysmography and venous outflow plethysmography have not been performed prior to the surgical intervention, which is another limitation of the study.

Clinically only one of our 35 patients has a C5 grade according to the CEAP classification. This is also nearly similar to other data. Wagenhäuser et al reported one patient with an active ulcer and one with a healed ulcer in a study cohort of 26 patients [1, 13]. Interestingly, only 31% of the patients had signs of chronic venous insufficiency (C3-C6) in this data. Concerning the Villalta score there were no signs of PTS in 43% of our patients and 46% of all participants had mild PTS. Notably, only 11% of our patients demonstrated moderate signs of PTS and no patient had a severe PTS. This is in contrast to others, who describe a proportion of rates without PTS up to 80% after surgical thrombectomy [13]. Concerning the PTS rates after endovascular thrombus removal, our results seem to be similar, as in the stratified analysis of the ATTRACT trial, PTS rates of 49% have been published [9, 11]. In the present study, 57% of all patients had a PTS, defined by a Villalta score > 5. Most patients had a mild sequel. Apparently, early thrombus removal strategies may not prevent PTS itself, but reduce severe forms and venous symptoms [11]. Nevertheless, a prospective study of surgical thrombectomy with a defined protocol and distinct follow-up examinations should address this question in detail, even though a prospective trial is not always feasible due to the limited number of patients per year.

According to the guidelines of the American venous forum, endovenous techniques are recommended as first line therapy [6]. The possibility of a percutaneous access to the deep venous system in local anesthesia is the advantage of an endovenous strategy, whereas, general anesthesia is indispensable during surgical thrombectomy. On the other hand active internal bleeding, trauma, recent cerebrovascular events or intracranial surgery, recent surgery within the last 10 days and pregnancy are absolute contraindications for catheter-directed thrombolysis. Therefore surgical thrombectomy should be favored in these aspects and venous vascular specialists should provide these skills. Further studies concerning surgical thrombectomy are necessary, even though endovenous methods have been recommended as first-line therapy.

## Conclusion

Postthrombotic syndrome constitutes a serious long-term complication of a deep venous thrombosis [2], especially if iliofemoral vein segments are involved. Patients with an acute, symptomatic iliofemoral deep vein thrombosis should be treated aggressively if they are candidates for early thrombus removing strategies [6]. This study demonstrates excellent patency rates and a good clinical hemodynamic outcome after a mean follow-up of 8.5 years after surgical thrombectomy of iliofemoral veins, even though venous refilling time and venous outflow are significantly lower in the treated legs when compared to the contralateral non-treated leg.

## Supporting information

S1 Table Measurement of venous hemodynamics. VO venous outflow, RT refilling time.
(PDF)
