# Supplementary material for: Surgical thrombectomy for iliofemoral deep vein thrombosis: Patient outcomes at 8.5 years
Source: PLoS One. 2020 Jun 18;15(6):e0235003. doi: 10.1371/journal.pone.0235003 (PMC7302664; doi:10.1371/journal.pone.0235003)
Supplement: S1 Table — VO venous outflow, RT refilling time. (PDF) [file pone.0235003.s001.pdf]

n	VO healthy	VO treated	RT healthy	RT treated
1	87,0	72,0	21,0	29,0
2	39,0	45,0	43,0	9,0
3	66,0	78,0	9,0	4,0
4	90,0	143,9	25,0	18,0
5				
6				
7	63,0	21,0	31,0	8,0
8	102,1	60,0	18,0	13,0
9	174,6	54,0	15,0	9,0
10	108,1	107,8	30,0	24,0
11	60,0	47,9	44,0	3,0
12	80,9	39,0	43,0	9,0
13	56,9	62,9	26,0	5,0
14	73,0	87,0	23,0	44,0
15	57,0	60,0	10,0	7,0
16	87,0	95,9	44,0	44,0
17	105,0	108,0	29,0	17,0
18	162,0	93,1	19,0	18,0
19	83,9	57,1	43,0	24,0
20	93,0	72,0	20,0	11,0
21	69,1	83,8	44,0	43,0
22	69,0	33,0	24,0	13,0
23	51,0	33,0	not usable	not usable
24	51,0	42,0	15,0	8,0
25	105,1	27,0	4,0	5,0
26	60,0	68,9	43,0	3,0
27	129,0	120,0	8,0	14,0
28				
29	54,0	48,0	11,0	25,0
30	45,0	68,9	43,0	43,0
31	90,0	93,0	26,0	26,0
32	24,0	15,0	20,0	4,0
33	63,0	75,0	18,0	7,0
34	93,0	57,0	31,0	11,0
35	101,9	45,0	15,0	8,0
